# Alternative Splicing of Toll-Like Receptor 9 Transcript in Teleost Fish Grouper Is Regulated by NF-κB Signaling via Phosphorylation of the C-Terminal Domain of the RPB1 Subunit of RNA Polymerase II

**DOI:** 10.1371/journal.pone.0163415

**Published:** 2016-09-22

**Authors:** Frank Fang-Yao Lee, Cho-Fat Hui, Tien-Hsien Chang, Pinwen Peter Chiou

**Affiliations:** 1 Molecular and Biological Agricultural Sciences, Taiwan International Graduate Program, Academia Sinica, Taipei, Taiwan; 2 Genomic Research Center, Academia Sinica, Taipei, Taiwan; 3 Graduate Institute of Biotechnology, National Chung-Hsing University, Taichung, Taiwan; 4 Institute of Cellular and Organismic Biology, Academia Sinica, Taipei, Taiwan; 5 Biotechnology Center, National Chung-Hsing University, Taichung, Taiwan; 6 Department of Aquaculture, National Taiwan Ocean University, Keelung, Taiwan; University of Hyderabad, INDIA

## Abstract

Similar to its mammalian counterparts, teleost Toll-like receptor 9 (TLR9) recognizes unmethylated CpG DNA presented in the genome of bacteria or DNA viruses and initiates signaling pathway(s) for immune responses. We have previously shown that the TLR9 pathway in grouper, an economically important teleost, can be debilitated by an inhibitory gTLR9B isoform, whose production is mediated by RNA alternative splicing. However, how does grouper TLR9 (gTLR9) signaling impinge on the RNA splicing machinery to produce *gTlr9B* is unknown. Here we show that the *gTlr9* alternative splicing is regulated through ligand-induced phosphorylation of the C-terminal domain (CTD) of the largest subunit of RNA polymerase II (Pol II). We first observed that ligand-activated NF- κB pathway biased the production of the *gTlr9B* isoform. Because NF- κB is known to recruit p-TEFb kinase, which phosphorylates the Pol II CTD at Ser2 residues, we examined p-TEFb’s role in alternative splicing. We found that promoting p-TEFb kinase activity significantly favored the production of the *gTlr9B* isoform, whereas inhibiting p-TEFb yielded an opposite result. We further showed that p-TEFb-mediated production of the *gTlr9B* isoform down-regulates its own immune responses, suggesting a self-limiting mechanism. Taken together, our data indicate a feedback mechanism of the gTLR9 signaling pathway to regulate the alternative splicing machinery, which in turn produces an inhibitor to the pathway.

## Introduction

Toll-like receptors (TLRs) play important roles in innate immune system by recognizing pathogen associated molecular pattern (PAMP) that are found exclusively in microorganisms and transducing signals to initiate corresponding responses. Fish TLRs, in general, are similar to their counterparts in mouse and human. As fish are water-living organisms, the threats encountered are different from those of mice or human. In addition, fish possess a less advanced adaptive immune system compared to that in mammals. Therefore, it is not surprising that an expanded repertoire of TLRs and function exist to detect fish-specific PAMP or compensate for the deficiency in adaptive immunity. For example, genes encoding major histocompatibility complex (*MHC*) class II, *CD4*, and invariant chain (*Ii*) in Atlantic cod (*Gadus morhua*) are lost. To compensate for the deficiency of adaptive branches, it is suggested that expanded reservoir of MHC class I and *Tlr* genes are used [1]. The complexity of fish innate molecules also leads to unique regulatory mechanisms to control their immune responses. For example, zebrafish Tlr3, whose activation also lead to NF-kB pathway, does not utilize the conserved interaction etween Trif and Traf6 [2]. Another example is that rainbow trout uses soluble Tlr5 to positive regulate the signaling pathway. This isoform has been shown to augment human TLR5 signaling *in-vitro* [3].

Toll-like receptor 9 (TLR9) recognizes synthetic CpG oligodeoxynucleotides (CpG ODN) or unmethylated CpG motifs in the genomes of bacteria or DNA viruses [4, 5]. After binding to the ligand, TLR9 interacts with the adaptor protein myeloid differentiation primary response 88 (MyD88) through their C-terminal toll/interleukin1 receptor (TIR) domains. MyD88 bridges TLR9 to the downstream signaling kinase IL-1 receptor associated kinase 4 (IRAK4). IRAK4 then phosphorylates IRAK1, allowing IRAK1 to interact with TNF receptor associated factor 6 (TRAF6) to assemble into a signaling complex. These series of events ultimately lead to the activation of nuclear factor kappa B (NF-κB) and activator protein-1 (AP-1) that regulate the transcription of proinflammatory cytokines such as IL-1β[6].

The signaling pathway initiated by TLR is an important step to mount immune response. However, acutely overreacted or chronically activated immune responses may cause diseases such as sepsis, atherosclerosis, and cancer [7, 8]. To prevent this, mechanisms to attenuate TLR signaling pathways are necessary. One of the known mechanisms is to produce inhibitory isoforms via RNA alternative splicing. Alternatively spliced isoforms are commonly found in the TLR signaling. By estimate, 256 alternatively spliced transcripts encompassing receptors, adaptors and signaling molecules in the TLR cascade have been identified [9]. For example, the mouse soluble Toll-like receptor 4 (smTLR4), which is an alternatively spliced TLR4 isoform, has been reported to serve as a decoy and negatively regulates lipopolysaccharide (LPS)-induced NF-κB and tumor necrosis factor (TNF) signaling in RAW264.1 cells [10]. MyD88s, an alternatively spliced isoform of MyD88 adaptor protein, cannot bridge the downstream protein IRAK4 for NF-κB activation [11, 12]. The IRAK1 signaling protein has three additional alternatively spliced isoforms, IRAK1b, IRAK1-S, and IRAK1c, none of them have kinase activity but the former two retain the ability to induce NF-κB activation. Conversely, IRAK1c acts as an inhibitor in the TLR4 signaling in response to LPS [13, 14].

Previous studies in our laboratory on grouper *Tlr9* is another example of aforementioned inhibitory isoforms that limit the immune responses. Groupers (*Epinephelus spp*.) are warm-water marine fish species of great economic importance. Grouper *Tlr9* gene encodes two distinct mRNA isoforms, namely *gTlr9A* and *gTlr9B* [15]. gTLR9A protein contains all elements in a typical TLR, including an N-terminal leucine-rich-repeats (LRR) domain, a transmembrane region and a C-terminal TIR domain. *gTlr9B* is a ligand-induced alternatively spliced transcript, which retains the second intron that gives rise to a premature stop codon. Therefore, gTLR9B differs from gTLR9A in having a truncated TIR domain and, as such, functions as a negative regulator in the grouper TLR9 signaling [15]. While the importance of alternatively spliced isoforms as immune regulators is widely recognized, little is known about the mechanism of regulating mRNA alternative splicing in immune system. It has been shown infection with *E*.*coli* in human dendritic cells results in global impacts on alternative splicing events associated with cell development, endocytosis, antigen presentation and cell cycle arrest are identified. In addition, the expression of mRNA level of at least 70 splicing factors alter in these infected cells[16]. During T cell activation, the surface glycoprotein CD6 undergoes alternative splicing presumably through the inability to recruit serine/arginine-rich splicing factor 1 (SRSF1) [17]. Another example is that splicing factor SF3a plays a role in inhibiting alternatively spliced MyD88s production, thereby promoting inflammation [18]. Despite the examples above, one important question remaining poorly addressed is how the upstream immune signal is transduced to the splicing machinery to regulate the alternative splicing process. Because immune responses often involve in turning on a specific subset of genes, a role of RNA polymerase II (Pol II) as a mediator for transducing the upstream signaling to the downstream splicing is warranted a serious deliberation.

Growing evidence has supported a conceptual framework that transcription, mRNA splicing, and other RNA processing processes, such as mRNA export and 3’-end processing, are functionally coupled [19–27]. The idea of coupling posits that specific steps within this network can mutually influence each other in terms of reaction rate and production efficiency. Coupling of RNA processing to transcription is mediated through the C-terminal domain (CTD) of RPB1, the largest subunit of Pol II. CTD serves as a landing pad for factors required for transcription/RNA processing to interact with and brings them to close vicinity to nascent RNAs emerging from Pol II. CTD consists of 52 copies of YSPTSPS repeats and undergoes extensive post-translational modifications, such as phosphorylation and acetylation, during transcription cycle. Differential phosphorylation on the Pol II CTD Ser5 or Ser2 residues contributes to the association/disassociation of transcription/RNA processing factors that regulate different stages of transcription [28–30]. Two working models, recruitment coupling and kinetic coupling, have been proposed to explain the molecular tethering between transcription and splicing. The former involves in functional or physical association of different splicing factors to the Pol II CTD, thus resulting in regulation of splice-site choices [31]. The latter suggests that the Pol II elongation rate may dictate the availability of competing splicing sites or other *cis*-information thus affecting the outcome of alternative splicing [32].

In this study, we show that in grouper TLR9 signaling, ligand-induced NF-κB activation leads to phosphorylation of Pol II CTD Ser2, thereby biasing the *gTlr9* alternative splicing for increasing production of the negative regulator gTLR9B as a means of self-limiting response. In addition, we demonstrate that this signaling-dependent alternative splicing strategy also functions in grouper macrophage that expresses endogenous *gTlr9*, thereby attesting to its physiological relevance.

## Material and Methods

### Cells and animals

Grouper kidney cells (GK cells) were a gift from Dr. Yu-Shen Lai of the National Yilan University and were kept in Leibovitz’s L-15 media supplement with 10% fetal bovine serum (FBS) at 28°C [33]. Orange-spotted grouper (*Epinephelus coioides*) were obtained from the National Cheng Kung University and kept in a 40-ton holding tank at a density of 40 fish per tank. Constant aeration and fresh seawater circulation were provided from holding tanks. The animal handling and experimental procedure were approved by the Institutional Animal Care and Use Committee (IACUC) of the Academia Sinica under approval protocol# RFiZOOCP2008094.

For enriching grouper macrophages, head kidney tissues were collected from eight fish euthanized with tricaine methanesulfonate (Sigma Aldrich, St. Louis, MO). Collected head-kidney tissues were ground by passing through 100-μm Cell Strainer (Thermo Fisher, Waltham, MA). The ground tissues were then layered on a 30–50% Percoll solution and centrifuged at 400 RPM for 40 min at 4°C. A distinct band containing grouper macrophage cells were formed and collected to a fresh 15-ml centrifuge tube. After extensive washing with a fresh serum-free L-15 medium, the grouper macrophages were seeded onto 6-well plates at the density of approximately 1X10^5^ cells/well with the L-15 medium containing 10% FBS.

### Cloning and analysis of *gTlr9* promoter sequence

Genomic DNA isolated from GK cells were used for cloning the *gTlr9* promoter sequence with Genome Walker Universal kit (Clontech, Mountain View, CA). Briefly, isolated genomic DNA was subjected to restriction enzyme digestion followed by ligating to an adaptor. Oligonucleotide set specific to the adaptor and *gTlr9* coding region were used to amplify fragment containing sequence upstream transcription start site (TSS) and partial known *gTlr9* coding sequence. PCR amplified fragments were then cloned into pJET1.2 vector (CloneJET PCR cloning kit, Thermo Fisher) for later sequencing. TSS upstream sequences were analyzed by softberry-N-site (http://goo.gl/SI81dj) for transcription factor binding sites prediction.

### Plasmid construction and transfection

Two plasmids encoding either human RNA polymerase II large subunit (RPB1) wild type or RPB1 with slow elongation mutation (R749H, C4 mutant) are gifts kindly provided by Dr. A. Kornblihtt, University of Buenos Aires. Both RPB1 plasmids contain an additional N792D mutation that confers resistance to α-aminitin [34, 35]. Plasmid encodes RPB1 CTD 1–25 (RPB1_1-25_) was constructed by in-frame deletion of Spe1 sites between WT RPB1 CTD residue 26 and carboxyl terminus [36]. YFP tagged NF-kB was constructed by in-frame inserting PCR-amplified NF-kB fragment into Xho1/BamH1 sites of pEYFP-C1 (Clontech). All plasmids were verified by sequencing before further experiment. Plasmids transfection was performed with Lipofectamin 2000 (Thermo Fisher), following the manufacturer’s instructions. To select cells transfected with human RPB1, L-15 medium containing α-aminitin at the concentration of 5 μg/ml were added to the transfected cells 24 hours after transfection.

### *gTlr9* agonist and inhibitor assays

A custom-designed CpG ODN was purchased from Sigma Aldrich to stimulate the grouper TLR9 signaling. Briefly, 5X10^5^ GK cells per well were seeded onto 6-well plates. After GK cells were fully attached to the plate, fresh serum free L-15 medium were replenished to avoid any interference from serum for at least 4 hours. CpG ODN diluted in a serum free L-15 medium at the final concentration of 500 nM were later add to the cells to stimulate gTLR9 signaling pathway. For inhibitor assay, 5,6-Dichloro-1-β-D-ribofuranosylbenzimidazole (DRB, Sigma), camptothecin (CPT, Sigma), BAY 11–7082 (Sigma), and SR11302 (Tocris Bioscience, Bristol, UK) were dissolved in DMSO and diluted into a serum free L-15 medium at the concentration of 20 μg/ml, 4 μg/ml, 20 nM and 100 nM, respectively. PMA (Phorbol myristate acetate, Sigma) activation was performed by adding PMA into a serum free L-15 medium at final concentration of 10 nM. Drug-treated cells or CpG ODN-stimulated cells were harvested for RNA extraction at indicated time points.

### RNA isolation and analysis of RNA samples

**Fig 1:**
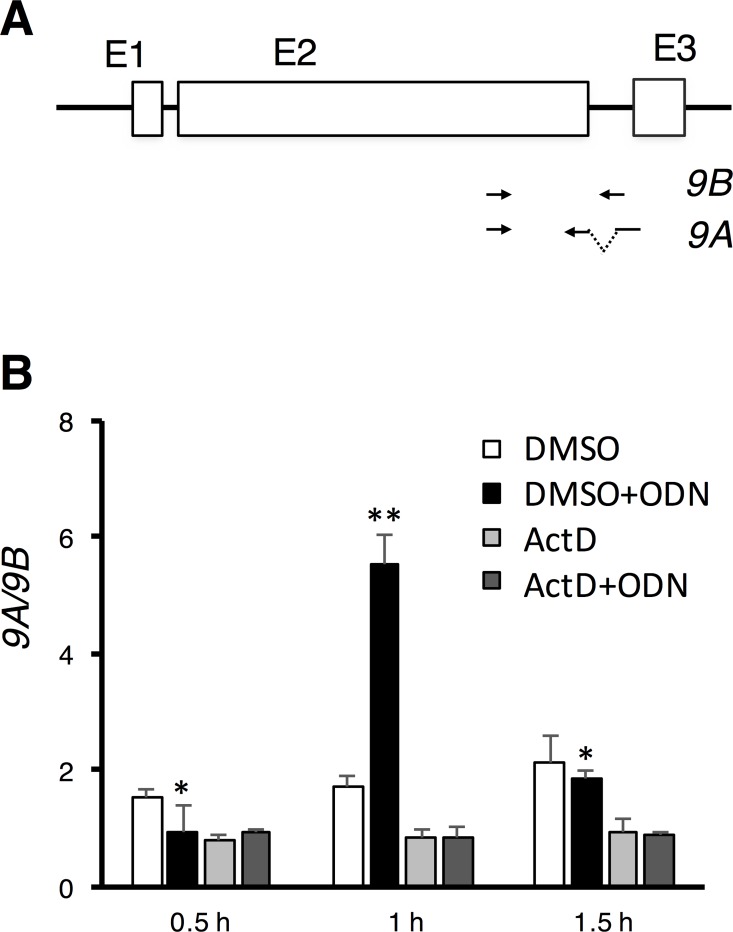
*gTlr9* alternative splicing requires active transcription. (**A**) The *gTlr9* gene is consisted of three exons (E1, E2, and E3) separated by two introns. Oligonucleotide primer pairs used to specifically amplify *9A* and *9B* are illustrated below. (**B**) Actinomycin D (ActD) shutdown of transcription abolished ligand-induced *gTlr9* alternative splicing. DMSO, solvent for dissolving ActD; ODN, stimulation ligand. The *9A*/*9B* ratio was calculated by first normalizing *9A* and *9B* values against that of the ß-actin transcript. Upon ODN stimulation, the *9A*/*9B* ratio initially increased (~1 h) but then dropped (Cf. black bars and white bars at all three time points). In contrast, under ActD inhibition, the *9A*/*9B* ratio remained the same. Results are presented as the mean ± SD (n = 3). Student’s t-test, 0.01< p < 0.05 (*) and p < 0.01 (**).

Total RNAs from the GK cells or macrophages were harvested with RNAzol reagents (Molecular Research Center, Cincinnati, OH) following manufacturer’s protocol. All the RNAs were checked for remaining *gTlr9* genomic DNA contamination with PCR against constitutively spliced intron I region. For reverse transcription, 3 μg of total RNAs isolated from cells were converted to cDNA with Roche Transcriptor First Strand cDNA synthesis Kit (Roche, Indianapolis, IN) with oligo dT primer. Oligonucleotide sets that base-paired with exon II-exon III junction was used to specifically detect the *gTlr9A* while primer that base-paired intron II was used to detect the *gTlr9B* in qPCR analysis as illustrated in Fig 1A. The qPCR reaction was conducted with 0.5 μM of each oligonucleotide sets in 1X SYBR green PCR master mix (LabStar, Taipei, Taiwan) with following thermocycler setting: 95°C 2 minutes followed by 40 cycles of 95°C 15 seconds and 60°C for 60s. Each sample was performed in triplicate and normalized expression to ß-actin. Oligonucleotides used in the study were listed in S1 Table.

### Chromatin-immunoprecipitate (ChIP) assay

ChIP assay was carried out with Millipore EZ-Magna ChIP A/G Chromatin Immunoprecipitation Kit (Millipore, Billerica, MA). Briefly, 1x10^7^ cells were fixed with 1% formaldehyde and then lysed with lysis buffer to isolate DNAs. Isolated DNAs were sheared into ~300 base pairs fragments with Biruptor Pico sonicator (Diagenode, Denville, NJ). Chromatin-immunoprecipitation was performed with the following antibodies: anti-total Pol II CTD clone 8WG16 (abcam), anti-serine 2 Pol II clone H5 (abcam) and anti-serine 5 Pol II (abcam). After extensive wash, DNAs were removed from bounded Pol II by proteinase K treatment and then subjected to qPCR analysis. Oligonucleotides sets were designed to amplify *gTlr9* promoter region (-1714 to -1654, compare to transcription start site), exon I (1206 to 1251), middle of exon II (1944 to 1996), before alternate intron II (3383 to 3422) and exon III (3710 to 3748). Oligonucleotide sequences are also listed in supplement S1 Table. ChIP results were presented as the average of the percentage of immunoprecipitate sample DNA to 5% input DNA in triplicate qPCR reactions.

### Western blot and ELISA assay

To detect Pol II phosphorylation under drug treatment, total proteins from GK cells were extracted with RIPA buffer (Millipore) and resolved on 6% SDS page. After transferring to PVDF membrane, Pol II was probed with antibody against total Pol II (clone N20, Santa Cruz, Dallas, TX), Pol II CTD P-ser2 phosphorylation clone H5 (abcam, Cambridge, UK), and Pol II CTD P-ser5 phosphorylation (abcam). For detecting IL-1B expression, 100 μl culture medium from each time points and treatments were collected and the antigen was detected with a rabbit anti-grouper IL-1β antibody prepared in-house [15]. The results of the ELISA assay were acquired using a Varioskan Flash spectral scanning multimode plate reader (ThermoScientific).

## Results

### Active transcription is required for regulating *gTlr9* alternative splicing

We have previously observed that the ratio of the two alternatively spliced *gTlr9* isoforms, i.e., *9A* and *9B*, changes over time in response to ligand stimulation, with the ratio of *9A*/*9B* rises soon after stimulation and then declines [15]. Because splicing is known to be tightly linked to transcription, whose functional state can affect the outcome of alternative splicing, we speculate that the observed change of ratio of *9A*/*9B* may be regulated at the transcription level. To test this hypothesis, we first shut down transcription by actinomycin D (ActD) in GK cells and then examined the change of *9A*/*9B* ratio upon CpG ODN stimulation. We used two sets of oligonucleotide-primer pair to monitor the steady-state levels of *9A* and *9B* separately by real-time PCR (Fig 1A). We have previously shown that, under ActD treatment, the amount of *9A* and *9B* remained stable for at least 3 hours [15]. Therefore, the *9A/9B* ratio depends on the differential expression of isoforms rather than on possible selective degradation during the assay time course (~1.5 hr). Without addition of ActD, the *9A*/*9B* ratio rose (1 h post-stimulation) and then declined (1.5 h) as expected (Fig 1B). When transcription was turned off by ActD, the *9A*/*9B* ratio remained constant over the course of ligand stimulation. Likewise, the *9A*/*9B* ratio in the control experiments, i.e., without ligand and ActD or just ActD addition, were largely unchanged. These data thus lend the support that transcription is involved in governing the *9A*/*9B* ratio upon ligand stimulation.

### Activation of NF-κB favors production of the *9B* isoform

**Fig 2:**
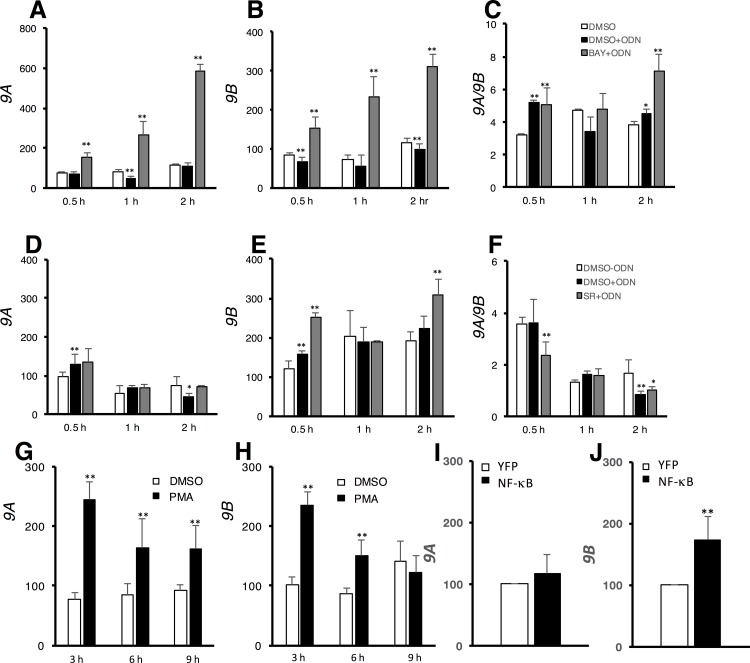
NF-κB regulates *gTlr9* alternative splicing. Two inhibitors BAY and SR, which targeted either NF-κB or AP-1 transcription factors, respectively, were applied under CpG ODN stimulation. The inhibition of one transcription factor under stimulation would result in activation of the other (see text for details). The expression of either *9A* or *9B* were determined by RT-qPCR and normalized against that of ß-actin as in Fig 1. Inhibition of NF-κB under ODN stimulation showed elevated *9A*, *9B* and *9A/9B* ratio (gray bars in each panel) (**2A-C**). In contrast, inhibition of AP-1 under stimulation selectively increased the expression of *9B* only, thus decreased *9A*/*9B* ratio (gray bars in each panel) (**2D-F**). (**2G-H**) Both *9A* and *9B* expression level increased in GK cells stimulated with PMA. (**2I**-**J)** YFP and YFP-tagged NF-κB were transfected into GK cells. 24 hours after transfection, relative expression of both *9A* and *9B* were assayed by RT-qPCR. The expression of *9A* remained largely the same (I), but 9B expression increased as compared to YFP vector control (J). Results are presented as the mean ± SD (n = 3). Student’s t-test, 0.01< p < 0.05 (*) and p < 0.01 (**).

It is known that gTLR9 ligand stimulation activates two inducible transcription factors, NF-κB and AP-1, which in turn trigger the transcription of their target genes. We therefore wondered whether these two transcription factors have a role in the observed transcriptionally regulated alternative splicing (Fig 1). This hypothesis was further strengthened by the finding of the NF-κB and AP-1 binding sites present upstream of the *gTlr9* gene. To test this hypothesis, we first used two specific inhibitors, SR 11302 and BAY 11–7082, to inhibit AP-1 and NF-κB respectively, and then placed the cells under continuous CpG ODN ligand stimulation. Our rationale was that inhibiting one transcription factor (e.g., AP-1) would result in sole activation of the other transcription factor (e.g., NF-κB), or *vice versa*. We found that the steady-state levels of *9A* (Fig 2A) and *9B* (Fig 2B) both increased over time upon AP-1 activation and the *9A*/*9B* ratio was also slightly elevated, which most likely reflected the intrinsic higher expression level of *9A* over *9B*. We interpret these data as AP-1 is responsible for regulating *gTlr9* transcription principally. This conclusion was further supported by an observation that, when AP-1 was activated by PMA, both the levels of *9A* and *9B* increased (Fig 2G and 2H). In contrast, when NF-κB was activated, the *9A* level remained largely the same (Fig 2D), but the level of *9B* increased (Fig 2E), resulting a prominent drop of the *9A*/*9B* ratio (Fig 2F). Likewise, the increase of *9B* level was observed when overexpressing YFP-tagged NF-κB in GK cells (Fig 2I and 2J). Taken together, these data suggest that activation of NF-κB favors an intron-retention event, thus yielding a lower *9A*/*9B* ratio.

### NF-κB regulates *gTlr9* alternative splicing through RNA polymerase II CTD phosphorylation

Our data suggest that NF-κB activation by gTLR9 ligand stimulation can bias *gTlr9* alternative splicing toward *9B*. To further confirm that CpG ODN stimulation indeed activated NF-κB pathway via 9A, we transfected 9A along with a NF-κB promoter-driven luciferase reporter plasmid into human 293T cells. The 293T cells do not express endogenous TLR9A, hence the observed luciferase activity induced by CpG ODN depends on ectopic expression 9A. We observed that luciferase activity increased in 9A-transfected, but not empty vector-transfected cells upon CpG ODN stimulation (S1 Fig). The results demonstrate that CpG ODN stimulation activated NF-κB pathway in a 9A-dependent manner.

**Fig 3:**
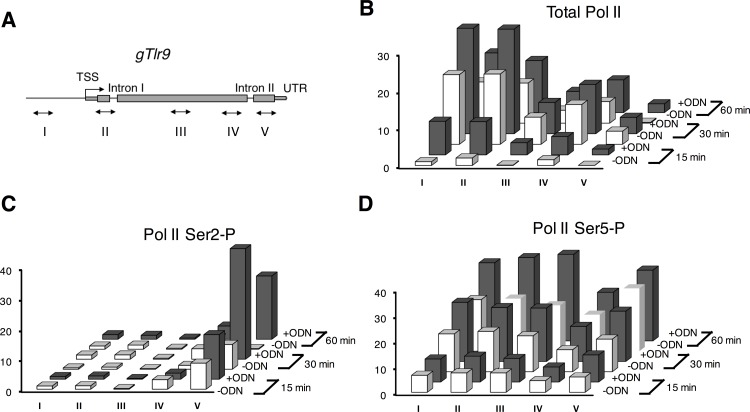
Increasing Pol II occupancy on *gTlr9* gene after ODN stimulation. (**A**) Oligonucleotide sets used in ChIP assay for various regions of the *gTlr9* gene are marked in Roman numerals. TSS, transcription start site; UTR, 3’ untranslated region. Occupancy of total Pol II (**B**), Pol II-CTD-Ser2 phosphorylated (Ser2-P) (**C**), and Pol II-CTD-Ser5 phosphorylated (Ser5-P) (**D**) on the *gTlr9* gene. Results are presented as the mean of triplicate samples. See Materials and methods for calculation of the percentage (vertical axis) of the ChIP data.

Previous studies suggest that the Pol II CTD phosphorylation state can impact on the outcome of alternative splicing [37–39]. Because NF-κB is known to recruit p-TEFb kinase for phosphorylating the second serine (Ser2) within the 52 hepta-peptide repeats of the CTD [40]. We therefore inspected CTD phosphorylation state of the transcribing Pol II on the *gTlr9* gene under ligand stimulation by ChIP analysis (Fig 3A). To this end, three different monoclonal antibodies were employed to immunoprecipitate Ser2-phosphorylated, Ser5- phosphorylated, and total Pol II, respectively. As expected, ligand stimulation increased both Ser2-phosphorylated and Ser5- phosphorylated Pol II, as well as total Pol II occupancy (Fig 3). This result suggests that both the transcription of the *gTlr9* gene and Pol II phosphorylation increase in response to ligand stimulation.

**Fig 4:**
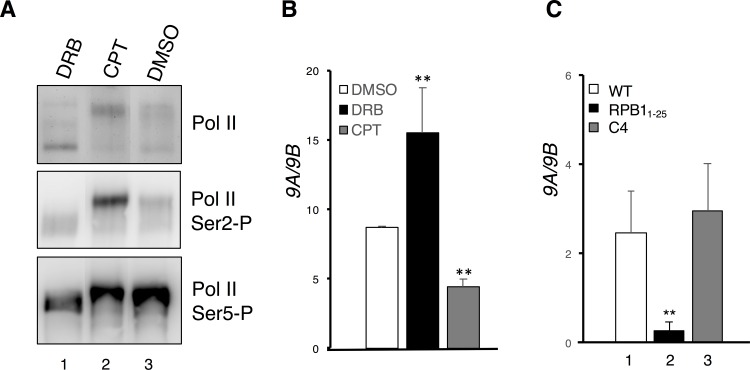
Pol II CTD phosphorylation and truncation impact on *gTlr9* alternative splicing. (**A**) CTD phosphorylation was examined by Western immunoblotting using specific monoclonal antibodies against different forms of Pol II. DRB, a p-TEFb inhibitor, blocks Ser2 phosphorylation (Ser2-P) and CPT promotes Ser2-P (Cf. lanes 1 and 2 vs. lane 3; top and middle panels). No significant changes were observed on Ser5-P in both treatments (bottom panel). (**B**) *9A/9B* ratio was calculated under drug treatments. DRB significantly elevates *9A*/*9B* ratio, whereas CPT decreases *9A*/*9B* ratio. (**C**) Impacts on the *9A*/*9B* ratio by Pol II variants. WT, Pol II containing a full-length RPB1; RPB1_1-25_, Pol II harboring a truncated form of RPB1 in which the 26–52 repeats at its CTD are deleted; C4, Pol II carrying an R749H alteration within RPB1. All three RPB1 clones harbor an additional α-aminitin resistant mutation (N792D) for selection.

To directly test whether p-TEFb-mediated Pol II CTD phosphorylation has any consequence in regulating the *gTlr9* alternative splicing, we sought to perturb p-TEFb kinase activity by either DRB or CPT. DRB is a nucleoside analog commonly used to inhibit p-TEFb [41]; in contrast, CPT promotes Pol II CTD phosphorylation by releasing p-TEFb from 7SK snoRNP complex in which it resides as an inactive form [42]. Consistent with findings in previous studies [43], DRB treatment decreased Ser2 phosphorylation, thus converting Pol II into a hypophosphorylated state (top and middle panels, lane 1, Fig 4A). In contrast, CPT promoted CTD Ser2 hyperphosphorylation (top and middle panels, lane 2, Fig 4A). Neither DRB nor CPT treatment appeared to have any effect on Ser5 phosphorylation (bottom panel, Fig 4A). We then analyzed the alterative splicing pattern for *gTlr9* under the drug-treatment conditions. In the presence of DRB, the production of *9A* isoform was favored, whereas the *9B* isoform production was preferred in the presence of CPT (Fig 4B). These results thus indicate that p-TEFb-mediated phosphorylation on CTD Ser2 is critical to *gTlr9* alternative splicing.

Ideally, alteration of the many Ser2 residues on CTD would be preferable for another test of the role of Ser2 phosphorylation in *gTlr9* alternative splicing. However, this proves to be difficult. We thus asked whether truncation of CTD, which was predicted to grossly alter the CTD phosphorylation, may also impact on *gTlr9* alternative splicing. To this end, we constructed an α-aminitin-resistant RPB1 in which the 26–52 repeats of its CTD tail was deleted (thereafter RPB1_1-25_) [36, 37]. We then transfected the GK cells with plasmids encoding either wild-type RPB1 or RPB1_1-25_, which was followed by addition of α-aminitin to inhibit the endogenous Pol II activity. Mock-transfected GK cells died within two days after α-aminitin addition while α-aminitin-resistant RPB1-transfected GK cells could survive several passages in α-aminitin-containing medium for at least a month. The observation suggests that grouper GK cells can utilize human RPB1 to transcribe mRNAs. RT-qPCR analysis revealed that *gTlr9* alternative splicing strongly favored the production of *9B* over *9A*, when transcription was done by the RPB1_1-25_-containing Pol II (Fig 4C). These results again support a critical role of CTD in *gTlr9* alternative splicing (see Discussion).

Because *9B* is an intron-retention isoform, the observed *9B* preference may arise from impaired splicing machinery, instead of alternative splicing regulation by CTD. To further exclude the possibility, we PCR-amplified constitutively spliced *gTlr9* intron I sequence and found the intron I was efficiently spliced out under both WT and RPB1_1-25_-containing Pol II background (data not shown). The result suggests that truncation in Pol II CTD does not impair splicing machinery and the apparent *9B* preference is the result of alternative splicing regulation.

An alternative, although not necessarily mutually exclusive, model for explaining how transcription may impact on the splicing outcome is through kinetic coupling [32]. In addition, CPT and DRB are reported to interfere with the Pol II elongation rate [44, 45]. We therefore wished to examine whether slowing down transcription may alter the *gTlr9* alternative splicing pattern. To this end, we expressed a slow-elongating RPB1 mutant (C4 mutant) [32, 46] in GK cells and collected total RNA for RT-qPCR analysis. No significant change of alternative splicing outcome could be detected (Fig 4C). Likewise, when trichostatin A, a compound capable of accelerating Pol II elongation rate [47], was used, instead, change of the alternative splicing pattern was not observed either (data not shown). These data suggest that the kinetic coupling model is less likely to be involved in governing the *gTlr9* alternative splicing.

### Functional impact of the *gTlr9* alternative splicing in cell lines and enriched fish macrophages

**Fig 5:**
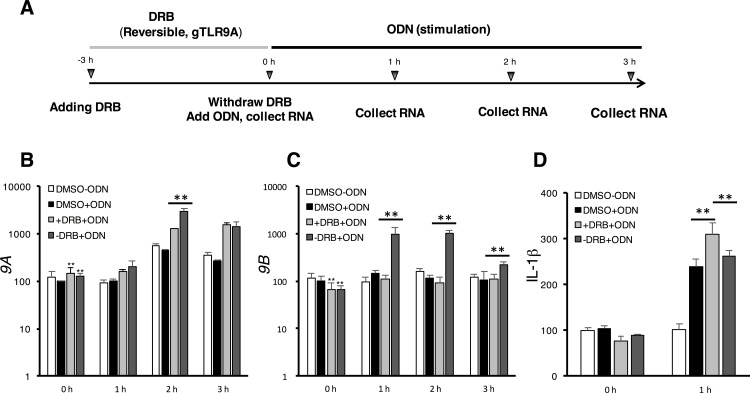
Self-limiting of the gTLR9 signaling by *gTlr9* alternative splicing. (**A**) Experiment design (see text for detailed description) (**B-C**) +DRB+ODN indicates that DRB was left in the culture medium throughout the course of ODN stimulation. In contrast,–DRB+ODN represents that DRB was withdrawn prior to ODN stimulation. Relative expression of *9A* and *9B* normalized to ß-actin was determined. DRB pre-treatment (0 h) led to slight increase of *9A* (**B**) and decrease of *9B* (**C**) (Cf. the two gray bars vs. white and black bars). Subsequent stimulation by ODN resulted in strong elevation of *9B* (1–3 h, dark gray bars) (**C**). (**D**) IL-1ß protein expression. The expression of IL-1ß in the DMSO group at 0 h was set as 100%. The expression pattern of IL-1ß at 1 h roughly correlated with the expression of *9A* and *9B*, where *9A* expression favored and *9B* expression suppressed IL-1ß production. Results are presented as the mean ± SD (n = 3). Student’s t-test, 0.01< p < 0.05 (*) and p < 0.01 (**).

The production of the *9A* and *9B* transcript isoforms have been reported in fish species closely related to grouper, although the underlying mechanism and its biological significance are not addressed [15, 48, 49]. This conservation of the alternative-spliced isoforms implies roles of both isoforms in immune regulation. Indeed, we have previously shown that the *9B-*encoded product, which is missing the signal-transducing TIR domain, behaves like a negative regulator of the corresponding immune pathway [15]. This and the fact that the *9A*/*9B* ratio rises soon after ligand stimulation and then declines [15] appear to be consistent with the notion that the TLR9 signaling can be attenuated after a successful immune response. We therefore hypothesize that alternative splicing is employed as an effective means to regulate the TLR9 signaling. To test this hypothesis, we first used DRB to promote the production of *9A* isoform over *9B* (see Fig 4B). After removing DRB, we stimulated the cells with the CpG ODN ligand (Fig 5A), which was expected to turn on the NF-κB pathway and lead to preferential production of the *9B* isoform and attenuation of the immune response. Indeed, after withdrawing DRB treatment and before ligand stimulation, the level of *9A* isoform slightly increased as expected, whereas the level of *9B* decreased (Cf. Fig 5B and 5C; 0 h time points). An hour after ligand stimulation, we observed that the level of *9B* dramatically increased (Fig 5C; 1 h time point), while the level of *9A* remained largely the same (Fig 5B; 1 h time point). At the latter time points, gradual increase of *9A* and decrease of *9B* were observed (Fig 5B and 5C), as if the system had returned to homeostasis. We also simultaneously monitored the immune response in the course of the experimentation (Fig 5D) by assaying the expression of downstream cytokine IL-1β. The IL-1β level was found to negatively correlate with the level of *9B*, consistent with our hypothesis.

**Fig 6:**
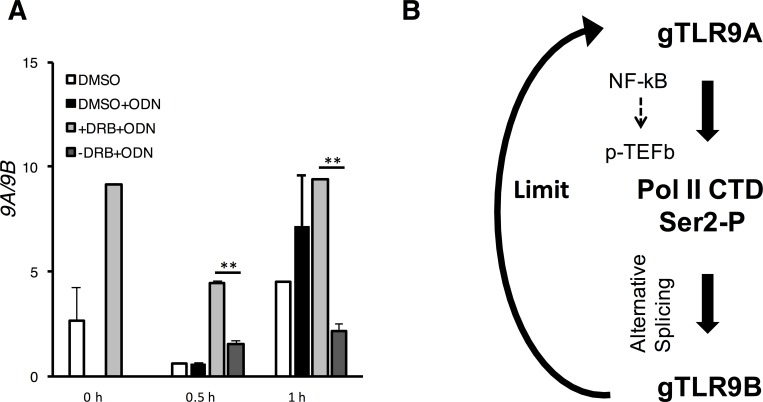
A working model for potential self-limiting regulation of *gTlr9* alternative splicing. (**A**) Enriched macrophage were pre-treated with DRB and stimulated with ODN with or without the removal of DRB as previously described in Fig 5. Increase of *9B* was observed from 0.5 h and beyond. (**B**) Proposed mechanism of *gTlr9* alternative splicing regulation.

To address the physiological importance of the *gTlr9* alternative splicing for immune regulation, we ask whether similar phenomenon exists in macrophages, immune cells that actively expressing TLR9. Macrophages were collected from 24 groupers as a pool and seeded into 6-well plates for DRB pretreatment and removal as described. After stimulation with CpG ODN, the *9A/9B* ratio decreased sharply after 0.5-h stimulation and remained low an hour later (Fig 6), a profile paralleled that of the GK-cells data (Fig 5). Taken altogether, our data suggest a role of NF-κB/p-TEFb-mediated phosphorylation of Pol II CTD in the regulation of grouper TLR9 pathway.

## Discussion

A common necessity of the TLR signaling pathway is to control the duration and magnitude of immune responses, so as to prevent prolong, improper-scaled, or unchecked responses [50, 51]. One strategy is to employ alternative splicing for producing a negative regulator to attenuate the signaling [52]. Consistent with this theme, we have previously shown that alternative splicing of *gTlr9* gene yields a negative regulator, gTLR9B, to down regulate the magnitude of gTLR9 responses [15]. However, in all cases of inhibitory isoforms reported [9–12, 14, 53], it is not clear how such alternative-splicing events are controlled. In this study, we focused on this issue by using grouper *Tlr9* gene as a model system and uncovered that NF-κB-mediated Pol II CTD phosphorylation regulated the alternative-splicing event in question. Hence, our finding uncovers a self-limiting mechanism of grouper TLR9 signaling pathway, in which the activation of the pathway eventually augments the alternative splicing event that produces an inhibitory isoform to subdue the pathway.

Previous studies on how signaling influences splicing have uncovered that the expression or the properties of specific RNA binding proteins are altered, leading to a desired alternative splicing outcome. For example, the Ras-pathway-induced alternative splicing of the CD44-v5 exon is achieved by using ERK kinase to modify Sam68 [54]. Alternatively, Mallory et al. [55] showed that, in T-cell signaling, activation of NF-κB regulates the activity of the splicing factor CELF2 through combined increases in transcription and mRNA stability. In this context, our findings indicate yet another pathway of NF-κB-signaling-regulated alternative splicing via, instead, direct modification of the Pol II CTD tail at Ser2 by p-TEFb. We noted that the response to stimuli in this case is rapid, in that a shift of the alternative splicing pattern can be readily observed within ~30 min (Fig 1). This observation suggests that in essence the gTLR9 signaling can be promptly terminated once the stimulus is removed.

In this study, we employed two different strategies to perturb phosphorylation of CTD Ser2 residues, i.e., chemical inhibition of p-TEFb by DRB (Fig 4B) and truncation of the CTD repeats (Fig 4C). Although both strategies are expected to reduce the overall level of Ser2 phosphorylation, they led to opposite outcomes, with DRB increasing and CTD truncation decreasing the *9A*/*9B* ratio, respectively. We offer the following possible explanations as to how this may have occurred. First, truncation of Pol II CTD may more severely disrupt the delicate balance of phosphorylation and dephosphorylation, which is required for Pol II to progress from initiation, elongation, to termination in an orderly manner [56]. Second, truncation of Pol II CTD may result in, at least in part, a loss of binding sites for factors participating in splicing regulation. Third, the two strategies are likely to differentially impact CTD Ser2 phosphorylation both in terms of the positions of affected Ser2 residues and the extent of phosphorylation loss. Regardless, our results clearly demonstrated the importance of Pol II CTD in regulating the *gTlr9* alternative splicing.

It seems highly plausible that the NF-κB-mediated alternative splicing mechanism for the *Tlr9* gene in grouper also functions in related fish species, given that the architecture of these *Tlr9* genes is essentially the same. A broader issue then is to consider whether the signaling-dependent CTD phosphorylation for regulating alternative splicing is also employed in other biological systems. Meta-analysis of a published report by Ip and colleagues [57] suggests that this may be the case (S2 Table). In this report, which aimed to examine the global effect of kinetic coupling, DRB and CPT were used to slow down Pol II elongation rate in PMA-stimulated Jurkat cells. Because DRB and CPT are also known to respectively inhibit or promote Pol II CTD phosphorylation [41, 42], we searched for alternative-splicing events that responded to DRB and CPT in an opposite manner. Among all the alternative splicing events in response to either DRB or CPT treatment, 92 were found to occur in both treatments. Out of these 92 events, 57 (62%) of them favored a particular alternative-splicing pattern, such as exon inclusion, consistent with the idea of a reduced Pol II elongation rate. Interestingly, an opposite alternative-splicing pattern was observed for the remaining 35 events (38%), e.g., exon inclusion by DRB treatment as oppose to exon skipping by CPT treatment. Among these 35 events, the case of interferon receptor 2 (IFNAR2) merits further consideration. Interferon receptor 2 interacts with Type I interferon to drive the transcription of interferon-stimulated genes through JAK-STAT signaling pathway. IFNAR2’s expression could be regulated via JAK-STAT signaling pathway through the action of STAT1, which has been reported to interact with p-TEFb [58]. Notably, DRB and CPT treatments resulted in an opposite effect on IFNAR2 in terms of its alternative splicing pattern, suggesting such a shift is mediated by p-TEFb, a scenario highly reminiscent of that of gTLR9 through the NF-κB pathway and p-TEFb. Should this turned out to be the case for IFNAR2, mechanistically speaking, a control of signaling-dependent alternative splicing regulation via the action of p-TEFb on Pol II CTD may prove to be a widespread and perhaps even evolutionarily conserved strategy.

## Conclusion

Together with our previous study, we propose here a mechanistic model to explain the temporal nature of gTLR9 signaling regulation in relationship to the Pol II-CTD-coupled alternative splicing. This model (Fig 6B) encompasses binding of ligands to gTLR9A to initiate signal-dependent CTD Ser2 phosphorylation through NF-κB and p-TEFb kinase. The resulting CTD phosphorylation then rapidly biases the *gTlr9* alternative splicing to produce *gTlr9B*. The gTLR9B, which is capable of ligand binding but cannot be assembled into signaling complex [15], would in turn acts as a molecular sink to down-regulate gTLR9 signaling. Although we have presented the impact of Pol II CTD phosphorylation on the *gTlr9* alternative splicing regulation, we cannot rule out the possibility that additional factors may also contribute to the process. For example, post-translational modifications, such as phosphorylation or acetylation, on splicing factor(s) are known to regulate alternative splicing [54, 59, 60]. It remains to be elucidated as to which splicing regulators are recruited to Ser2-phosphorylated Pol II CTD under the signaling stimulation condition. Works are in progress toward this important aim. Finally, from a perspective of vaccine development in which ODN has been used as adjuvant in grouper [61], it will be interesting to assay whether a more potent vaccination outcome can be achieved by modulating the alternative splicing event to remove the negatively-regulatory gTLR9B.

## Supporting Information

S1 Fig CpG ODN activates NF-κB signaling through transfected 9A in 293T cells. Plasmid encoding *9A* cDNA, NF-κB promoter driven firefly luciferase reporter plasmid and an internal control CMV-driven renilla luciferase plasmid were co-transfected into human 293T cells. At 24 hours post transfection, transfected 293T cells were stimulated with CpG ODN and collected for dual-luciferase activity assay. Firefly luciferase activities were further normalized to renilla luciferase. All values are presented in mean ± SD of triplicate samples.
(TIFF)

S1 Table Primers used in this study. (DOCX)

S2 Table Alternative spliced events in responses to DRB and CPT. “+”: exon inclusion;”-“: exon exclusion. DRB: 5,6-Dichloro-1-β-D-ribofuranosylbenzimidazole; CPT: camptothecin.
(DOCX)
