# Supplementary figures and images for: Alternative Splicing of Toll-Like Receptor 9 Transcript in Teleost Fish Grouper Is Regulated by NF-κB Signaling via Phosphorylation of the C-Terminal Domain of the RPB1 Subunit of RNA Polymerase II
Source: PLoS One. 2016 Sep 22;11(9):e0163415. doi: 10.1371/journal.pone.0163415 (PMC5033454; doi:10.1371/journal.pone.0163415)

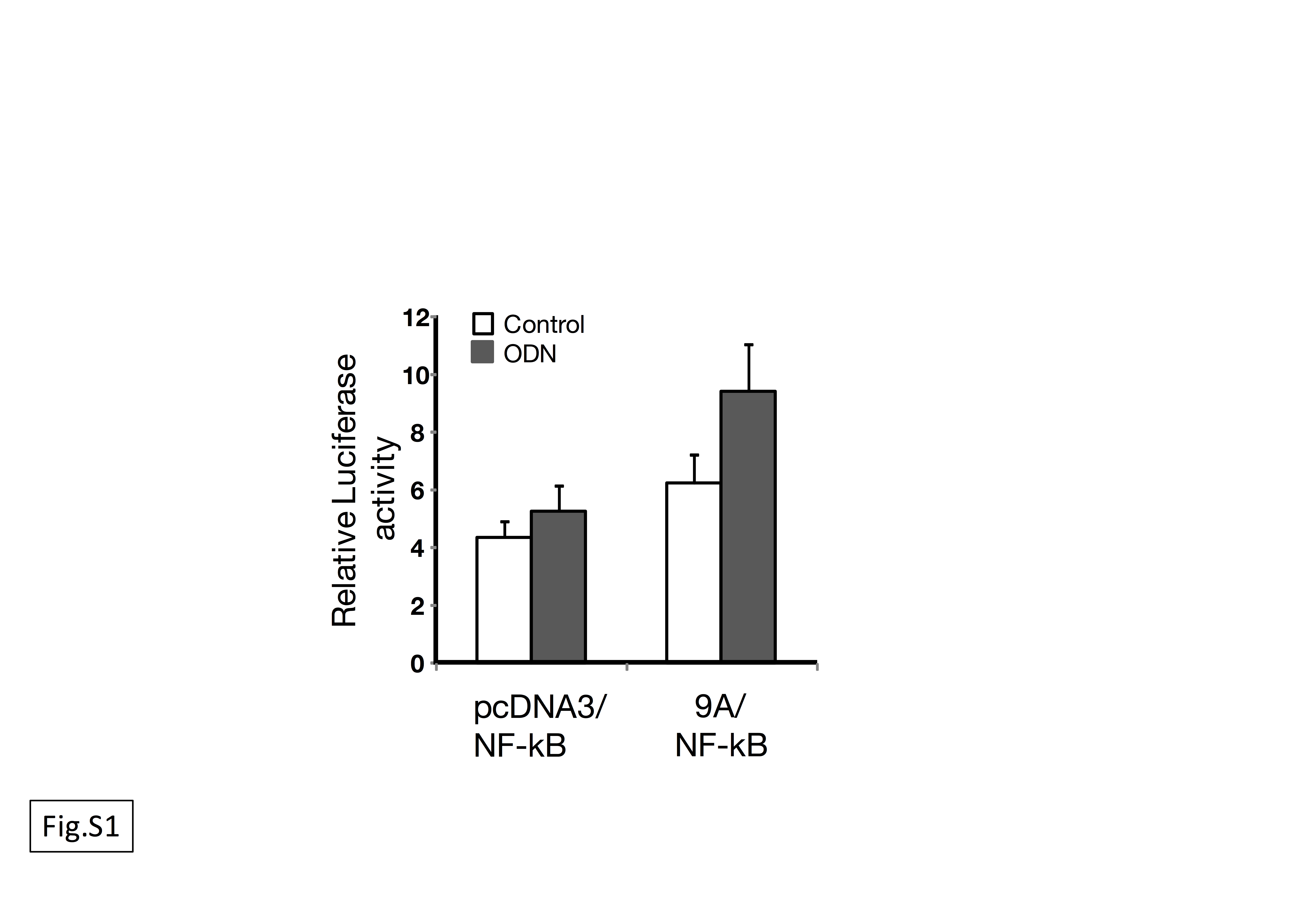

Supplement: S1 Fig — Plasmid encoding 9A cDNA, NF-κB promoter driven firefly luciferase reporter plasmid and an internal control CMV-driven renilla luciferase plasmid were co-transfected into human 293T cells. At 24 hours post transfection, transfected 293T cells were stimulated with CpG ODN and collected for dual-luciferase activity assay. Firefly luciferase activities were further normalized to renilla luciferase. All values are presented in mean ± SD of triplicate samples. (TIFF) [file pone.0163415.s001.tiff]
