# Supplementary material for: Alternative Splicing of Toll-Like Receptor 9 Transcript in Teleost Fish Grouper Is Regulated by NF-κB Signaling via Phosphorylation of the C-Terminal Domain of the RPB1 Subunit of RNA Polymerase II
Source: PLoS One. 2016 Sep 22;11(9):e0163415. doi: 10.1371/journal.pone.0163415 (PMC5033454; doi:10.1371/journal.pone.0163415)
Supplement: S1 Table — (DOCX) [file pone.0163415.s002.docx]

|  |  |  |
| --- | --- | --- |
| **Table S1** |  |  |
| **Primers used in this study** | |  |
|  |  |  |
| Primers for real-time PCR | |  |
|  | FWD | REV |
| *gTlr9A* | 5’-GTTTGTGCTGTCCAGCGGT-3’ | 5’GCATAGCTGCATCCACCTTCTC-3' |
| *gTlr9B* | 5’-GCGACTTCTGGACGAGAAGGT-3’ | 5’-AACATGGCTACAACAGGATATGAATC-3’ |
| *B-actin* | 5'-CCTGACAGAGCGTGGCTACTC-3' | 5’-CCTTGATGTCACGCACGATT-3’ |
|  |  |  |
| Primers for ChIP assay | |  |
|  | FWD | REV |
| 1 | 5'-CATCACAAATGTTCCCTGACAGTAT-3' | 5'-CGTCGCCTTTGAGAACACTTT-3' |
| 2 | 5'-TGGAACGTTTGGATTTAAGAGAGA-3' | 5'-TCAAGTTGGCAAATGCTCCAT-3' |
| 3 | 5'-CGAAAAGCTACATCTGGTAACGATAT-3' | 5'-TGTCCTCCCCCATTTCCA-3' |
| 4 | 5'-CAATCTGCATAACGCCGTGTA-3' | 5'-ACCGCTGGACAGCACAAAC-3' |
| 5 | 5'-TCTGTGTTATCCTGGCCGAAA-3' | 5'-TCTCATTCGGTTCCAGAAAAGG-3' |
