# Supplementary material for: Alternative Splicing of Toll-Like Receptor 9 Transcript in Teleost Fish Grouper Is Regulated by NF-κB Signaling via Phosphorylation of the C-Terminal Domain of the RPB1 Subunit of RNA Polymerase II
Source: PLoS One. 2016 Sep 22;11(9):e0163415. doi: 10.1371/journal.pone.0163415 (PMC5033454; doi:10.1371/journal.pone.0163415)
Supplement: S2 Table — “+”: exon inclusion;”-“: exon exclusion. DRB: 5,6-Dichloro-1-β-D-ribofuranosylbenzimidazole; CPT: camptothecin. (DOCX) [file pone.0163415.s003.docx]

**Table S2**

**Alternative Splicing Events (ASE) in respond to DRB and CPT**

| Increasing ASE in DRB | | |  | Increasing ASE in DRB | | |
| --- | --- | --- | --- | --- | --- | --- |
| Decreasing ASE in CPT | | |  | Increasing ASE in CPT | | |
| **ASE** | **Gene** | **Accessoin no.** |  | **ASE** | **Gene** | **Accessoin no.** |
| BBY123 | SPPL2A | BG188747 |  | BBY1195 | HMGCS1 | CN343489 |
| BBY1583 | PHLDB1 | AK125783 |  | BBY1383 | ODF2 | NM_002540 |
| BBY3165 | GNAO1 | BX647214 |  | BBY1504 | P4HA1 | AL574109 |
|  |  |  |  | BBY1542 | ATG13 | AI142351 |
| Decreasing ASE in DRB | | |  | BBY1555 | C11orf30 | BG534694 |
| Increasing ASE in CPT | | |  | BBY160 | M-RIP | AL834513 |
| **ASE** | **Gene** | **Accessoin no.** |  | BBY167 | EIF4A2 | BF306318 |
| BBY1341 | ATP6V1H | NM_213619 |  | BBY1737 | MCRS1 | AL559888 |
| BBY1616 | CLNS1A | CB044090 |  | BBY1764 | TPT1 | AA412025 |
| BBY1630 | FAM76B | BX647586 |  | BBY1786 | ZC3H14 | NM_207661 |
| BBY1703 | KDM2B | AK127328 |  | BBY181 | SNRPB | CA487896 |
| BBY173 | ADD3 | NM_016824 |  | BBY1811 | NIN | NM_020921 |
| BBY174 | PICALM | NM_007166 |  | BBY1835 | MEF2A | AL831995 |
| BBY1858 | UBE3A | CF994438 |  | BBY1913 | XPO6 | BF094888 |
| BBY1916 | BRD7 | BX377621 |  | BBY2 | IVNS1ABP (NS1BP) | AK023123 |
| BBY1937 | MPRIP | BC009982 |  | BBY2036 | SFRS2 (SC35) | AK092489 |
| BBY1948 | ARRB2 | AA357190 |  | BBY2347 | LRPPRC | AY289212 |
| BBY2106 | GMFG | AV714415 |  | BBY2459 | SND1 | AY273894 |
| BBY2176 | IFNAR2 | X89814 |  | BBY256 | SF3B3 | BG474021 |
| BBY2342 | APH1A | AY358951 |  | BBY257 | MAPT | NM_016835 |
| BBY2551 | TM9SF2 | U81006 |  | BBY268 | HMGXB4 | AL079310 |
| BBY292 | RUNX1 | D43969 |  | BBY274 | AGFG1 | BC030592 |
| BBY3120 | MAST1 | BC027985 |  | BBY33 | NT5C3 | BE958264 |
| BBY3216 | PAPOLA | X76770 |  | BBY3599 | U2AF1 | AL832665 |
| BBY3294 | ATP5C1 | BC026049 |  | BBY364 | FUBP1 | AI752038 |
| BBY3434 | LCP2 | BC016618 |  | BBY3739 | RCC2 | AL359612 |
| BBY3974 | FHL1 | BC010998 |  | BBY4110 | TMEM49 | AF214006 |
| BBY4194 | NAE1 | AY197612 |  | BBY4246 | LTBP4 | AF051345 |
| BBY4803 | ACTG1 | BC010417 |  | BBY48 | TCF12 | BE779382 |
| BBY4911 | KDM6A | AF000993 |  | BBY4981 | HNRPDL | AY453824 |
| BBY5266 | DOCK2 | D86964 |  | BBY5019 | YLPM1 | BC007792 |
| BBY546 | ARHGEF1 | U64105 |  | BBY5104 | RPL13A | AB082924 |
| BBY5486 | SSPO | AK123170 |  | BBY5452 | WDR33 | AK002156 |
| BBY550 | ILF3 | U10324 |  | BBY5478 | PNPLA6 | BC050553 |
| BBY559 | MTCH2 | BI465155 |  | BBY5653 | LGALS9 | AB006782 |
| BBY577 | CHR2SYT | AB033054 |  | BBY5708 | PDZD4 | AB040877 |
| BBY618 | KIAA0494 | AB007963 |  | BBY624 | HLA-G | M32800 |
| BBY911 | WDFY1 | BU949997 |  | BBY655 | HNRPLL | AK000155 |
| BBY975 | ATP2C1 | CN481726 |  | BBY675 | PTPRC | NM_080922 |
|  |  |  |  | BBY7 | EIF4G3 | BC030578 |
|  |  |  |  | BBY830 | ENO1 | CB266003 |
|  |  |  |  | BBY976 | RBM6 | CA488450 |
|  |  |  |  |  |  |  |
|  |  |  |  | Decreasing ASE in DRB | | |
|  |  |  |  | Decreasing ASE in CPT | | |
|  |  |  |  | **ASE** | **Gene** | **Accessoin no.** |
|  |  |  |  | BBY1228 | UBE2J1 | AI708167 |
|  |  |  |  | BBY1448 | CELF2 | BF901085 |
|  |  |  |  | BBY1730 | R3HDM2 | BF955291 |
|  |  |  |  | BBY177 | ACLY | BC006195 |
|  |  |  |  | BBY2277 | IL2RG | AB102796 |
|  |  |  |  | BBY2283 | SH3KBP1 | BX433376 |
|  |  |  |  | BBY2642 | HUWE1 | AB002310 |
|  |  |  |  | BBY2927 | ATP2B4 | M83363 |
|  |  |  |  | BBY5285 | HNRNPL | BC069184 |
|  |  |  |  | BBY5401 | AHCYL1 | BC010681 |
|  |  |  |  | BBY662 | SDCCAG3 | BC014515 |
|  |  |  |  | BBY738 | YBX1 | BF971939 |
|  |  |  |  | BBY791 | APH1A | BE935456 |
|  |  |  |  | BBY874 | HADHB | CB152624 |
|  |  |  |  | BBY996 | EIF4A2 | AL117412 |
